# “Emotional Intelligence, Psychological Distress, and Conflict Resolution Among Healthcare Professionals”

**DOI:** 10.1192/j.eurpsy.2024.1737

**Published:** 2024-08-27

**Authors:** M. Theodoratou, A. Papadopoulos

**Affiliations:** 1 Social Sciences, Hellenic Open University, Patras, Greece

## Abstract

**Introduction:**

Emotional Intelligence (EI) plays a substantial role in shaping the behavior, overall well-being, and performance of individuals. In the context of healthcare, where professionals frequently confront a demanding work environment, there is a notable prevalence of high Psychological Distress (PD). Consequently, conflicts are a recurrent phenomenon within healthcare settings, exerting impacts on healthcare professionals, patients, and their families.

**Objectives:**

Aims:

1. Investigate the link between Emotional Intelligence (EI) and conflict management among healthcare professionals.

2. Examine how Psychological Distress (PD) relates to conflict management in healthcare.

3. Explore age, specialization, and experience’s influence on EI dimensions.

4. Analyze EI’s impact on healthcare professionals’ conflict resolution choices.

5. Assess how demographics affect conflict resolution preferences among healthcare workers.

These aims explore EI, PD, demographics, and conflict management in healthcare, informing skill enhancement and improved conflict resolution practices.

**Methods:**

This study involved 143 healthcare professionals from diverse regions of Greece. Electronic surveys gathered demographic data and assessed Emotional Intelligence (via a dedicated questionnaire), Psychological Distress (using the Kessler K6+ questionnaire), and Conflict Resolution strategies.

**Results:**

The majority of participants were female (69.2%), with 42.7% aged 46-55 and 30.8% aged 36-45. Age was significantly associated with “Self-awareness” (P=0.032) and “Social Skills” (P=0.009 and 0.007) within Emotional Intelligence dimensions. Negative correlations emerged between Psychological Distress and Emotional Intelligence dimensions (-0.46 to -0.19). Additionally, Psychological Distress showed negative correlations with several Conflict Resolution dimensions: ‘Atmosphere’ (-0.20), ‘Doables’ (-0.28), ‘Mutual Benefit Agreements’ (-0.18), ‘Needs’ (-0.23), and ‘Extra Considerations’ (-0.27). Participants below 35 had higher scores in “Power” (p=0.002), while those aged 46 and above scored higher in “Options” (p=0.002 and 0.009) for conflict resolution.

**Conclusions:**

In summary, this study underscores EI’s relevance in healthcare, especially its influence on PD and conflict resolution. Developing EI competencies offers promise for improving healthcare professionals’ emotional well-being and conflict-handling abilities, ultimately benefiting patient care and staff satisfaction. Further research and tailored interventions are warranted to advance this field at an academic level.

**Disclosure of Interest:**

None Declared

